# 4-(2-Chloro­anilino)-3-phenyl­furan-2(5*H*)-one

**DOI:** 10.1107/S1600536811044308

**Published:** 2011-10-29

**Authors:** Zhu-Ping Xiao, Ze-Jun Huang, Xiao-Yang Liu, Kai-Shuang Xiang, She-Rong Yu

**Affiliations:** a College of Chemistry and Chemical Engineering, Jishou University, Jishou 416000, People’s Republic of China

## Abstract

The title compound, C_16_H_12_ClNO_2_, featuring a furan-2(5*H*)-one (γ-butyrolactone) core, contains two mol­ecules (*A* and *B*) in the asymmetric unit, with different dihedral angles between the central ring and the pendant phenyl and chloro­benzene rings [43.33 (8) and 20.16 (8)°, respectively, for *A*, and 47.79 (8) and 13.87 (8)°, respectively, for *B*]. In the crystal, the *A* mol­ecules are linked into [001] chains by single C—H⋯O inter­actions. The *B* mol­ecules also form [001] chains, but their relative orientations in the chains are quite different to those of the *A* mol­ecules so that adjacent *B* mol­ecules are linked by two C—H⋯O hydrogen bonds. Finally, C—H⋯O inter­actions and aromatic π–π stacking contacts [centroid–centroid separations = 3.754 (1) and 3.817 (1) Å] link the chains into a two-dimensional array parallel to (010).

## Related literature

For a related structure and background references, see: Xiao *et al.* (2011 ▶).
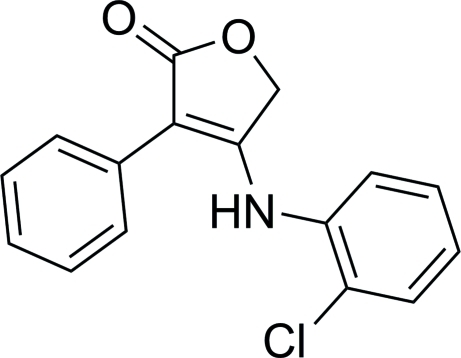


## Experimental

### 

#### Crystal data

C_16_H_12_ClNO_2_
*M* *_r_* = 285.72
Monoclinic, 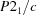

*a* = 7.7305 (5) Å
*b* = 27.4374 (18) Å
*c* = 12.6242 (9) Å
β = 92.145 (1)°
*V* = 2675.8 (3) Å^3^
*Z* = 8
Mo *K*α radiation
μ = 0.29 mm^−1^
*T* = 298 K
0.30 × 0.20 × 0.20 mm

#### Data collection

Bruker SMART APEX CCD diffractometer
Absorption correction: multi-scan (*SADABS*; Sheldrick, 1996 ▶) *T*_min_ = 0.919, *T*_max_ = 0.945
32744 measured reflections
6603 independent reflections
5086 reflections with *I* > 2σ(*I*)
*R*_int_ = 0.096

#### Refinement

*R*[*F* ^2^ > 2σ(*F* ^2^)] = 0.048
*wR*(*F* ^2^) = 0.144
*S* = 1.06
6603 reflections
370 parameters
H atoms treated by a mixture of independent and constrained refinement
Δρ_max_ = 0.36 e Å^−3^
Δρ_min_ = −0.30 e Å^−3^

### 

Data collection: *SMART* (Bruker, 2007 ▶); cell refinement: *SAINT* (Bruker, 2007 ▶); data reduction: *SAINT*; program(s) used to solve structure: *SHELXS97* (Sheldrick, 2008 ▶); program(s) used to refine structure: *SHELXL97* (Sheldrick, 2008 ▶); molecular graphics: *SHELXTL* (Sheldrick, 2008 ▶); software used to prepare material for publication: *SHELXL97*.

## Supplementary Material

Crystal structure: contains datablock(s) global, I. DOI: 10.1107/S1600536811044308/hb6469sup1.cif

Structure factors: contains datablock(s) I. DOI: 10.1107/S1600536811044308/hb6469Isup2.hkl

Supplementary material file. DOI: 10.1107/S1600536811044308/hb6469Isup3.cml

Additional supplementary materials:  crystallographic information; 3D view; checkCIF report

## Figures and Tables

**Table 1:** Hydrogen-bond geometry (Å, °) *Cg*2 is the centroid of the C1–C6 ring.

*D*—H⋯*A*	*D*—H	H⋯*A*	*D*⋯*A*	*D*—H⋯*A*
C16—H16⋯O1^i^	0.93	2.47	3.305 (2)	149
C31—H31⋯O4^ii^	0.93	2.58	3.422 (2)	151
C32—H32⋯O3^ii^	0.93	2.48	3.305 (2)	148
C19—H19⋯*Cg*2^iii^	0.93	2.84	3.553 (2)	134

Symmetry codes: (i) 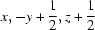; (ii) 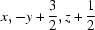; (iii) 
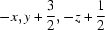
.

**Fig. 1.**
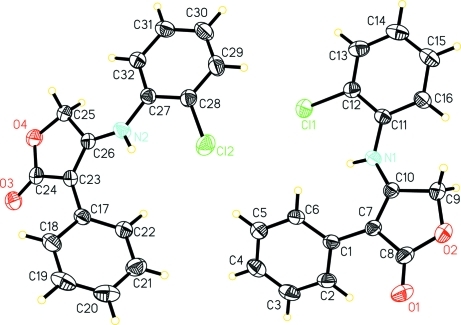
Molecular structure of the title compound. Displacement ellipsoids are drawn at the 30% probability level.

**Fig. 2.**
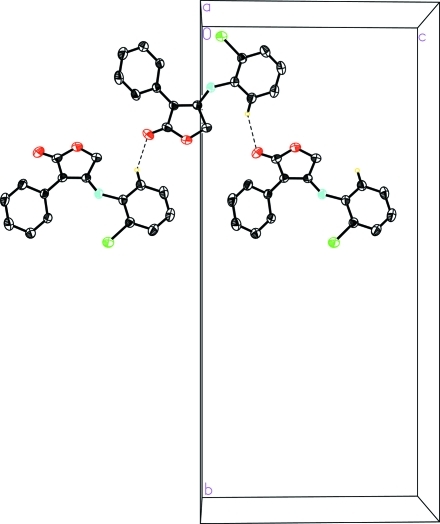
a. Molecules (A) form a one-dimensional line through intermolecular C—H···O hydrogen bonds. For the sake of clarity, the H atoms have been omitted except that involving in hydrogen bonds.

**Fig. 3.**
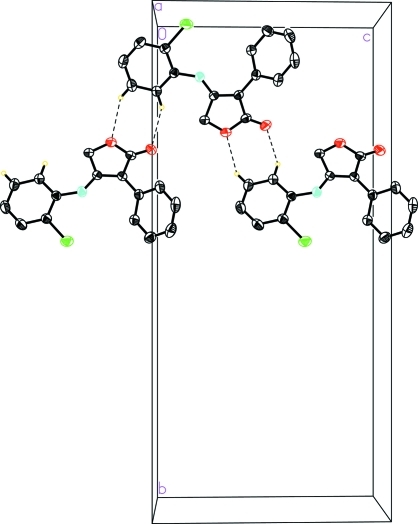
b. Molecules (B) form a one-dimensional line through intermolecular C—H···O hydrogen bonds. For the sake of clarity, the H atoms have been omitted except that involving in hydrogen bonds.

**Fig. 4.**
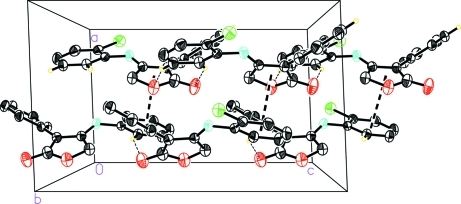
a. A two-dimensional sheet is formed by intermolecular C—H···π interactions and π–π contacts.

**Fig. 5.**
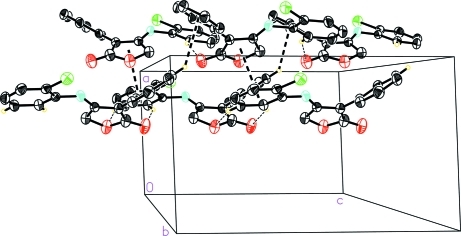
b. A two-dimensional sheet is formed by intermolecular C—H···π interactions and π–π contacts.

## supplementary crystallographic information

### Comment

As part of our ongoing studies of compounds with a γ-butyrolactone (furanone) core (Xiao *et al.*, 2011), we now report the structure of the title compound, (I).

The crystal structure of the title compound, 4-(2-chlorophenylamino)-3-phenylfuran-2(5*H*)-one, contains two crystallographically independent molecules (Fig. 1) in an asymmetric unit with difference of bond length lower than 0.009 Å. In molecule A (from C1 to C16, O1, O2, N1 and Cl1), the central furan-2(5*H*)-one ring make a dihedral angles of 43.33 (8) and 20.16 (8) ° with the benzene ring and the *o*-chloroaniline ring, repectively. While in the molecule B (from C17 to C32, O3, O4, N2 and Cl2), they are 47.79 (8) and 13.87 (8) °, respectively. For the convenience of description, molecular structure was discussed based on A. Bond distance C7—C10 (1.3490 (19) Å) is indicative of a double bond, and the title compound was therefore identified as a furan-2(5*H*)-one.

Interestingly, molecule A and molecule B show different interemolecular hydrogen bonding patterns. For molecule A, an infinite one-dimensional line is formed by C—H···O hydrogen bondings (Fig 2a). While molecules B are linked into a line by *R*^2^_2_(7) rings, which built from C31—H31···O4 and C32—H32···O3 hydrogen bonds centred at (0, 0, n) and (0, 0.0695, n) respectively, where n represents an integer (Fig. 2 b). Between the resulted lines, three kinds of intermolecular interactions were found and link molecules to further generate an infinite two-dimensional sheet. Of them, C—H···π occurs between C19 (in molecule B) and 3-benzene ring with "H···centroid" length of 2.840 Å, while π–π contacts occur between furanone rings and aniline rings with "centroid···centroid" lengths of 3.754 (1) and 3.817 (1) Å, respectively (Fig. 3a and Fig. 3 b).

### Experimental

To a methanol solution (20 ml) of 2-methoxy-1-naphthaldehyde (0.1 mmol, 17.4 mg) and 4-methylbenzohydrazide (0.1 mmol, 15.0 mg), a few drops of acetic acid were added. The mixture was refluxed for 1 h and then cooled to room temperature. The white crystalline solid was collected by filtration, washed with cold methanol and dried in air. Single crystals, suitable for X-ray diffraction, were obtained by slow evaporation of a methanol solution of the product in air.

### Refinement

The NH H-atom was located in a difference Fourier map and was refined with a distance restraint, N—H = 0.90 (1) \%A. The C-bound H atoms were positioned geometrically and refined using a riding model: C—H = 0.93 and 0.96 \%A, for CH and CH_3_ H-atoms, respectively, with *U*_iso_(H) = k × *U*_eq_(C) where k = 1.5 for CH_3_ H-atoms and k = 1.2 for all other H-atoms.

### Crystal data

**Table:**

C_16_H_12_ClNO_2_	*Z* = 8
*M**_r_* = 285.72	*F*(000) = 1184
Monoclinic, *P*2_1_/*c*	*D*_x_ = 1.418 Mg m^−^^3^
Hall symbol: -P 2ybc	Mo *K*α radiation, λ = 0.71073 Å
*a* = 7.7305 (5) Å	θ = 2.6–27.8°
*b* = 27.4374 (18) Å	µ = 0.29 mm^−^^1^
*c* = 12.6242 (9) Å	*T* = 298 K
β = 92.145 (1)°	Block, colorless
*V* = 2675.8 (3) Å^3^	0.30 × 0.20 × 0.20 mm

### Data collection

**Table:**

Bruker SMART APEX CCD diffractometer	6603 independent reflections
Radiation source: fine-focus sealed tube	5086 reflections with *I* > 2σ(*I*)
graphite	*R*_int_ = 0.096
φ and ω scan	θ_max_ = 28.3°, θ_min_ = 2.2°
Absorption correction: multi-scan (*SADABS*; Sheldrick, 1996)	*h* = −10→10
*T*_min_ = 0.919, *T*_max_ = 0.945	*k* = −36→36
32744 measured reflections	*l* = −16→16

### Refinement

**Table:**

Refinement on *F*^2^	Secondary atom site location: difference Fourier map
Least-squares matrix: full	Hydrogen site location: inferred from neighbouring sites
*R*[*F*^2^ > 2σ(*F*^2^)] = 0.048	H atoms treated by a mixture of independent and constrained refinement
*wR*(*F*^2^) = 0.144	*w* = 1/[σ^2^(*F*_o_^2^) + (0.080*P*)^2^ + 0.1033*P*] where *P* = (*F*_o_^2^ + 2*F*_c_^2^)/3
*S* = 1.06	(Δ/σ)_max_ = 0.001
6603 reflections	Δρ_max_ = 0.36 e Å^−^^3^
370 parameters	Δρ_min_ = −0.30 e Å^−^^3^
0 restraints	Extinction correction: *SHELXL97* (Sheldrick, 2008), Fc^*^=kFc[1+0.001xFc^2^λ^3^/sin(2θ)]^-1/4^
Primary atom site location: structure-invariant direct methods	Extinction coefficient: 0.0084 (10)

### Special details

**Table:**

Geometry. All e.s.d.'s (except the e.s.d. in the dihedral angle between two l.s. planes) are estimated using the full covariance matrix. The cell e.s.d.'s are taken into account individually in the estimation of e.s.d.'s in distances, angles and torsion angles; correlations between e.s.d.'s in cell parameters are only used when they are defined by crystal symmetry. An approximate (isotropic) treatment of cell e.s.d.'s is used for estimating e.s.d.'s involving l.s. planes.
Refinement. Refinement of *F*^2^ against ALL reflections. The weighted *R*-factor *wR* and goodness of fit *S* are based on *F*^2^, conventional *R*-factors *R* are based on *F*, with *F* set to zero for negative *F*^2^. The threshold expression of *F*^2^ > σ(*F*^2^) is used only for calculating *R*-factors(gt) *etc*. and is not relevant to the choice of reflections for refinement. *R*-factors based on *F*^2^ are statistically about twice as large as those based on *F*, and *R*- factors based on ALL data will be even larger.

### Fractional atomic coordinates and isotropic or equivalent isotropic displacement parameters (Å^2^)

**Table:**

	*x*	*y*	*z*	*U*_iso_*/*U*_eq_	
C1	0.27814 (18)	0.35945 (5)	0.29763 (10)	0.0461 (3)	
C2	0.3577 (2)	0.34203 (6)	0.20759 (12)	0.0583 (4)	
H2	0.3631	0.3086	0.1954	0.070*	
C3	0.4284 (2)	0.37386 (8)	0.13655 (13)	0.0682 (5)	
H3	0.4800	0.3618	0.0766	0.082*	
C4	0.4231 (2)	0.42301 (7)	0.15360 (13)	0.0684 (5)	
H4	0.4722	0.4442	0.1058	0.082*	
C5	0.3450 (2)	0.44111 (6)	0.24147 (13)	0.0609 (4)	
H5	0.3413	0.4746	0.2529	0.073*	
C6	0.27233 (19)	0.40972 (5)	0.31272 (11)	0.0504 (3)	
H6	0.2188	0.4223	0.3715	0.060*	
C7	0.20474 (18)	0.32524 (5)	0.37337 (11)	0.0475 (3)	
C8	0.1029 (2)	0.28279 (5)	0.34135 (13)	0.0608 (4)	
C9	0.1273 (2)	0.28158 (5)	0.52194 (13)	0.0578 (4)	
H9A	0.2096	0.2607	0.5599	0.069*	
H9B	0.0365	0.2906	0.5691	0.069*	
C10	0.21487 (17)	0.32582 (5)	0.48027 (11)	0.0454 (3)	
C11	0.28478 (17)	0.37210 (5)	0.64825 (10)	0.0457 (3)	
C12	0.33208 (18)	0.41894 (5)	0.68118 (11)	0.0500 (3)	
C13	0.3164 (2)	0.43397 (7)	0.78420 (13)	0.0656 (4)	
H13	0.3484	0.4655	0.8038	0.079*	
C14	0.2536 (2)	0.40257 (8)	0.85839 (13)	0.0729 (5)	
H14	0.2403	0.4129	0.9278	0.088*	
C15	0.2108 (2)	0.35592 (7)	0.82878 (13)	0.0681 (5)	
H15	0.1711	0.3343	0.8791	0.082*	
C16	0.2257 (2)	0.34051 (6)	0.72529 (12)	0.0561 (4)	
H16	0.1959	0.3087	0.7069	0.067*	
C17	0.22903 (18)	0.63216 (5)	0.06184 (11)	0.0487 (3)	
C18	0.1337 (2)	0.64534 (7)	−0.02958 (12)	0.0626 (4)	
H18	0.1155	0.6781	−0.0452	0.075*	
C19	0.0664 (2)	0.61005 (9)	−0.09697 (13)	0.0758 (6)	
H19	0.0023	0.6193	−0.1575	0.091*	
C20	0.0925 (2)	0.56142 (9)	−0.07617 (15)	0.0782 (6)	
H20	0.0468	0.5379	−0.1223	0.094*	
C21	0.1860 (2)	0.54794 (7)	0.01279 (15)	0.0692 (5)	
H21	0.2038	0.5150	0.0273	0.083*	
C22	0.2544 (2)	0.58261 (5)	0.08146 (12)	0.0554 (4)	
H22	0.3183	0.5728	0.1416	0.066*	
C23	0.29904 (19)	0.66934 (5)	0.13540 (11)	0.0490 (3)	
C24	0.3918 (2)	0.71252 (6)	0.10214 (13)	0.0621 (4)	
C25	0.3729 (2)	0.71710 (5)	0.28216 (12)	0.0559 (4)	
H25A	0.2888	0.7379	0.3150	0.067*	
H25B	0.4661	0.7101	0.3335	0.067*	
C26	0.29004 (18)	0.67104 (5)	0.24190 (11)	0.0458 (3)	
C27	0.21703 (17)	0.62944 (5)	0.41166 (11)	0.0451 (3)	
C28	0.16217 (18)	0.58432 (5)	0.44911 (12)	0.0499 (3)	
C29	0.1665 (2)	0.57327 (6)	0.55559 (14)	0.0648 (4)	
H29	0.1286	0.5430	0.5783	0.078*	
C30	0.2268 (2)	0.60706 (7)	0.62796 (13)	0.0708 (5)	
H30	0.2321	0.5996	0.6999	0.085*	
C31	0.2794 (2)	0.65192 (7)	0.59359 (13)	0.0640 (4)	
H31	0.3190	0.6750	0.6428	0.077*	
C32	0.2744 (2)	0.66332 (6)	0.48734 (12)	0.0548 (4)	
H32	0.3098	0.6940	0.4658	0.066*	
Cl1	0.40795 (6)	0.460028 (15)	0.58859 (4)	0.06829 (15)	
Cl2	0.09240 (6)	0.540051 (15)	0.35831 (4)	0.06933 (16)	
N1	0.30072 (17)	0.35959 (5)	0.54159 (10)	0.0512 (3)	
N2	0.21099 (18)	0.63755 (5)	0.30234 (10)	0.0553 (3)	
O1	0.05550 (19)	0.26896 (4)	0.25415 (10)	0.0813 (4)	
O2	0.05665 (17)	0.25763 (4)	0.42950 (10)	0.0711 (3)	
O3	0.4329 (2)	0.72534 (4)	0.01522 (10)	0.0833 (4)	
O4	0.43819 (18)	0.73964 (4)	0.18943 (10)	0.0716 (3)	
H1	0.354 (2)	0.3800 (6)	0.5078 (13)	0.054 (4)*	
H2A	0.164 (2)	0.6156 (6)	0.2687 (14)	0.061 (5)*	

### Atomic displacement parameters (Å^2^)

**Table:**

	*U*^11^	*U*^22^	*U*^33^	*U*^12^	*U*^13^	*U*^23^
C1	0.0511 (7)	0.0492 (7)	0.0377 (6)	0.0074 (6)	−0.0023 (5)	−0.0037 (5)
C2	0.0671 (9)	0.0633 (9)	0.0442 (8)	0.0156 (7)	0.0002 (7)	−0.0096 (7)
C3	0.0691 (10)	0.0927 (13)	0.0437 (8)	0.0134 (9)	0.0127 (7)	−0.0060 (8)
C4	0.0713 (10)	0.0840 (13)	0.0506 (9)	−0.0060 (9)	0.0108 (8)	0.0071 (8)
C5	0.0717 (10)	0.0574 (9)	0.0538 (8)	−0.0063 (7)	0.0037 (7)	0.0001 (7)
C6	0.0591 (8)	0.0521 (8)	0.0401 (7)	0.0035 (6)	0.0037 (6)	−0.0043 (6)
C7	0.0551 (8)	0.0409 (7)	0.0464 (7)	0.0075 (5)	0.0001 (6)	−0.0039 (5)
C8	0.0805 (11)	0.0430 (8)	0.0585 (9)	0.0052 (7)	−0.0026 (8)	−0.0088 (7)
C9	0.0736 (10)	0.0434 (8)	0.0565 (9)	−0.0004 (7)	0.0014 (7)	0.0026 (6)
C10	0.0498 (7)	0.0411 (7)	0.0455 (7)	0.0071 (5)	0.0028 (6)	−0.0014 (5)
C11	0.0437 (7)	0.0534 (8)	0.0399 (7)	0.0039 (5)	0.0008 (5)	−0.0001 (6)
C12	0.0482 (7)	0.0535 (8)	0.0481 (7)	0.0027 (6)	−0.0014 (6)	−0.0021 (6)
C13	0.0693 (10)	0.0725 (11)	0.0545 (9)	0.0040 (8)	−0.0057 (7)	−0.0160 (8)
C14	0.0790 (11)	0.0965 (14)	0.0434 (8)	0.0055 (10)	0.0022 (8)	−0.0121 (9)
C15	0.0726 (10)	0.0881 (13)	0.0440 (8)	0.0045 (9)	0.0078 (7)	0.0088 (8)
C16	0.0619 (9)	0.0595 (9)	0.0472 (8)	0.0006 (7)	0.0053 (6)	0.0040 (7)
C17	0.0500 (7)	0.0552 (8)	0.0411 (7)	0.0031 (6)	0.0045 (6)	0.0018 (6)
C18	0.0627 (9)	0.0801 (11)	0.0451 (8)	0.0156 (8)	0.0036 (7)	0.0056 (8)
C19	0.0630 (10)	0.1212 (18)	0.0428 (8)	0.0052 (10)	−0.0054 (7)	−0.0056 (10)
C20	0.0736 (11)	0.1020 (16)	0.0588 (10)	−0.0176 (10)	0.0018 (9)	−0.0241 (10)
C21	0.0794 (11)	0.0623 (10)	0.0662 (11)	−0.0134 (8)	0.0068 (9)	−0.0119 (8)
C22	0.0615 (9)	0.0550 (9)	0.0493 (8)	−0.0023 (7)	−0.0016 (7)	−0.0003 (6)
C23	0.0588 (8)	0.0424 (7)	0.0458 (7)	0.0057 (6)	0.0017 (6)	0.0049 (6)
C24	0.0901 (12)	0.0408 (8)	0.0559 (9)	0.0046 (7)	0.0109 (8)	0.0076 (7)
C25	0.0767 (10)	0.0398 (7)	0.0513 (8)	−0.0004 (6)	0.0055 (7)	0.0003 (6)
C26	0.0509 (7)	0.0409 (7)	0.0454 (7)	0.0032 (5)	−0.0005 (6)	0.0032 (5)
C27	0.0447 (7)	0.0465 (7)	0.0442 (7)	0.0031 (5)	0.0024 (5)	0.0026 (5)
C28	0.0472 (7)	0.0479 (8)	0.0550 (8)	0.0042 (6)	0.0071 (6)	0.0025 (6)
C29	0.0677 (10)	0.0620 (10)	0.0655 (10)	0.0057 (8)	0.0139 (8)	0.0197 (8)
C30	0.0814 (11)	0.0846 (12)	0.0469 (9)	0.0096 (10)	0.0076 (8)	0.0123 (8)
C31	0.0689 (10)	0.0760 (11)	0.0469 (8)	0.0049 (8)	−0.0009 (7)	−0.0058 (8)
C32	0.0633 (9)	0.0528 (8)	0.0482 (8)	0.0000 (6)	−0.0008 (6)	0.0000 (6)
Cl1	0.0816 (3)	0.0550 (3)	0.0685 (3)	−0.01316 (18)	0.0057 (2)	0.00081 (18)
Cl2	0.0762 (3)	0.0517 (2)	0.0803 (3)	−0.01332 (18)	0.0058 (2)	−0.00368 (19)
N1	0.0619 (7)	0.0526 (7)	0.0397 (6)	−0.0100 (6)	0.0081 (5)	−0.0016 (5)
N2	0.0683 (8)	0.0554 (7)	0.0419 (6)	−0.0165 (6)	−0.0028 (6)	0.0001 (5)
O1	0.1134 (10)	0.0628 (7)	0.0665 (8)	−0.0094 (7)	−0.0115 (7)	−0.0203 (6)
O2	0.0986 (9)	0.0456 (6)	0.0688 (7)	−0.0136 (6)	−0.0024 (7)	−0.0031 (5)
O3	0.1370 (12)	0.0537 (7)	0.0611 (7)	−0.0046 (7)	0.0279 (8)	0.0119 (6)
O4	0.1118 (10)	0.0415 (6)	0.0624 (7)	−0.0131 (6)	0.0159 (7)	0.0007 (5)

### Geometric parameters (Å, °)

**Table:**

C1—C6	1.393 (2)	C17—C22	1.395 (2)
C1—C2	1.3968 (19)	C17—C23	1.469 (2)
C1—C7	1.469 (2)	C18—C19	1.378 (3)
C2—C3	1.379 (3)	C18—H18	0.9300
C2—H2	0.9300	C19—C20	1.373 (3)
C3—C4	1.367 (3)	C19—H19	0.9300
C3—H3	0.9300	C20—C21	1.364 (3)
C4—C5	1.375 (2)	C20—H20	0.9300
C4—H4	0.9300	C21—C22	1.379 (2)
C5—C6	1.380 (2)	C21—H21	0.9300
C5—H5	0.9300	C22—H22	0.9300
C6—H6	0.9300	C23—C26	1.3497 (19)
C7—C10	1.3490 (19)	C23—C24	1.455 (2)
C7—C8	1.455 (2)	C24—O3	1.2063 (19)
C8—O1	1.208 (2)	C24—O4	1.367 (2)
C8—O2	1.368 (2)	C25—O4	1.4323 (19)
C9—O2	1.4296 (19)	C25—C26	1.4972 (19)
C9—C10	1.495 (2)	C25—H25A	0.9700
C9—H9A	0.9700	C25—H25B	0.9700
C9—H9B	0.9700	C26—N2	1.3547 (18)
C10—N1	1.3637 (18)	C27—C32	1.394 (2)
C11—C16	1.393 (2)	C27—C28	1.3966 (19)
C11—C12	1.395 (2)	C27—N2	1.3969 (18)
C11—N1	1.3995 (18)	C28—C29	1.377 (2)
C12—C13	1.374 (2)	C28—Cl2	1.7419 (16)
C12—Cl1	1.7415 (15)	C29—C30	1.371 (3)
C13—C14	1.374 (3)	C29—H29	0.9300
C13—H13	0.9300	C30—C31	1.372 (3)
C14—C15	1.371 (3)	C30—H30	0.9300
C14—H14	0.9300	C31—C32	1.377 (2)
C15—C16	1.382 (2)	C31—H31	0.9300
C15—H15	0.9300	C32—H32	0.9300
C16—H16	0.9300	N1—H1	0.824 (16)
C17—C18	1.393 (2)	N2—H2A	0.815 (17)
			
C6—C1—C2	117.86 (14)	C19—C18—H18	119.8
C6—C1—C7	121.89 (12)	C17—C18—H18	119.8
C2—C1—C7	120.25 (13)	C20—C19—C18	121.00 (16)
C3—C2—C1	120.65 (16)	C20—C19—H19	119.5
C3—C2—H2	119.7	C18—C19—H19	119.5
C1—C2—H2	119.7	C21—C20—C19	119.37 (17)
C4—C3—C2	120.50 (15)	C21—C20—H20	120.3
C4—C3—H3	119.7	C19—C20—H20	120.3
C2—C3—H3	119.7	C20—C21—C22	120.64 (18)
C3—C4—C5	120.00 (16)	C20—C21—H21	119.7
C3—C4—H4	120.0	C22—C21—H21	119.7
C5—C4—H4	120.0	C21—C22—C17	120.84 (15)
C4—C5—C6	120.14 (16)	C21—C22—H22	119.6
C4—C5—H5	119.9	C17—C22—H22	119.6
C6—C5—H5	119.9	C26—C23—C24	107.68 (13)
C5—C6—C1	120.84 (14)	C26—C23—C17	128.47 (13)
C5—C6—H6	119.6	C24—C23—C17	123.84 (13)
C1—C6—H6	119.6	O3—C24—O4	120.29 (15)
C10—C7—C8	107.34 (13)	O3—C24—C23	130.52 (16)
C10—C7—C1	129.37 (13)	O4—C24—C23	109.15 (13)
C8—C7—C1	123.29 (13)	O4—C25—C26	104.26 (12)
O1—C8—O2	120.20 (15)	O4—C25—H25A	110.9
O1—C8—C7	130.33 (17)	C26—C25—H25A	110.9
O2—C8—C7	109.45 (13)	O4—C25—H25B	110.9
O2—C9—C10	104.48 (12)	C26—C25—H25B	110.9
O2—C9—H9A	110.9	H25A—C25—H25B	108.9
C10—C9—H9A	110.9	C23—C26—N2	125.37 (13)
O2—C9—H9B	110.9	C23—C26—C25	109.27 (12)
C10—C9—H9B	110.9	N2—C26—C25	125.29 (13)
H9A—C9—H9B	108.9	C32—C27—C28	116.88 (13)
C7—C10—N1	125.78 (13)	C32—C27—N2	124.67 (13)
C7—C10—C9	109.41 (12)	C28—C27—N2	118.45 (13)
N1—C10—C9	124.69 (12)	C29—C28—C27	121.94 (15)
C16—C11—C12	117.02 (13)	C29—C28—Cl2	118.97 (12)
C16—C11—N1	124.21 (14)	C27—C28—Cl2	119.06 (11)
C12—C11—N1	118.76 (13)	C30—C29—C28	119.78 (15)
C13—C12—C11	121.81 (15)	C30—C29—H29	120.1
C13—C12—Cl1	119.00 (13)	C28—C29—H29	120.1
C11—C12—Cl1	119.17 (11)	C29—C30—C31	119.56 (16)
C12—C13—C14	120.18 (17)	C29—C30—H30	120.2
C12—C13—H13	119.9	C31—C30—H30	120.2
C14—C13—H13	119.9	C30—C31—C32	120.96 (17)
C15—C14—C13	119.17 (16)	C30—C31—H31	119.5
C15—C14—H14	120.4	C32—C31—H31	119.5
C13—C14—H14	120.4	C31—C32—C27	120.86 (15)
C14—C15—C16	121.04 (17)	C31—C32—H32	119.6
C14—C15—H15	119.5	C27—C32—H32	119.6
C16—C15—H15	119.5	C10—N1—C11	130.87 (13)
C15—C16—C11	120.73 (16)	C10—N1—H1	114.2 (12)
C15—C16—H16	119.6	C11—N1—H1	113.3 (12)
C11—C16—H16	119.6	C26—N2—C27	131.72 (13)
C18—C17—C22	117.82 (14)	C26—N2—H2A	114.2 (13)
C18—C17—C23	120.94 (14)	C27—N2—H2A	113.2 (13)
C22—C17—C23	121.24 (13)	C8—O2—C9	109.19 (12)
C19—C18—C17	120.33 (17)	C24—O4—C25	109.52 (12)
			
C6—C1—C2—C3	0.2 (2)	C23—C17—C22—C21	179.00 (14)
C7—C1—C2—C3	−179.41 (14)	C18—C17—C23—C26	132.18 (16)
C1—C2—C3—C4	0.6 (2)	C22—C17—C23—C26	−47.3 (2)
C2—C3—C4—C5	−0.8 (3)	C18—C17—C23—C24	−47.0 (2)
C3—C4—C5—C6	0.1 (3)	C22—C17—C23—C24	133.49 (16)
C4—C5—C6—C1	0.7 (2)	C26—C23—C24—O3	178.08 (19)
C2—C1—C6—C5	−0.8 (2)	C17—C23—C24—O3	−2.6 (3)
C7—C1—C6—C5	178.78 (13)	C26—C23—C24—O4	0.26 (18)
C6—C1—C7—C10	−42.7 (2)	C17—C23—C24—O4	179.59 (13)
C2—C1—C7—C10	136.91 (16)	C24—C23—C26—N2	178.94 (14)
C6—C1—C7—C8	137.21 (15)	C17—C23—C26—N2	−0.3 (2)
C2—C1—C7—C8	−43.2 (2)	C24—C23—C26—C25	1.93 (17)
C10—C7—C8—O1	176.81 (18)	C17—C23—C26—C25	−177.36 (14)
C1—C7—C8—O1	−3.1 (3)	O4—C25—C26—C23	−3.30 (17)
C10—C7—C8—O2	−1.51 (17)	O4—C25—C26—N2	179.68 (13)
C1—C7—C8—O2	178.58 (13)	C32—C27—C28—C29	−0.9 (2)
C8—C7—C10—N1	179.36 (13)	N2—C27—C28—C29	179.05 (14)
C1—C7—C10—N1	−0.7 (2)	C32—C27—C28—Cl2	−178.93 (11)
C8—C7—C10—C9	3.25 (16)	N2—C27—C28—Cl2	1.05 (18)
C1—C7—C10—C9	−176.85 (13)	C27—C28—C29—C30	−0.3 (2)
O2—C9—C10—C7	−3.77 (16)	Cl2—C28—C29—C30	177.69 (13)
O2—C9—C10—N1	−179.94 (13)	C28—C29—C30—C31	1.2 (3)
C16—C11—C12—C13	−2.0 (2)	C29—C30—C31—C32	−0.8 (3)
N1—C11—C12—C13	178.63 (14)	C30—C31—C32—C27	−0.5 (3)
C16—C11—C12—Cl1	179.71 (10)	C28—C27—C32—C31	1.3 (2)
N1—C11—C12—Cl1	0.30 (18)	N2—C27—C32—C31	−178.65 (15)
C11—C12—C13—C14	0.4 (2)	C7—C10—N1—C11	160.68 (15)
Cl1—C12—C13—C14	178.70 (13)	C9—C10—N1—C11	−23.8 (2)
C12—C13—C14—C15	1.5 (3)	C16—C11—N1—C10	26.5 (2)
C13—C14—C15—C16	−1.7 (3)	C12—C11—N1—C10	−154.17 (14)
C14—C15—C16—C11	0.0 (3)	C23—C26—N2—C27	166.70 (15)
C12—C11—C16—C15	1.8 (2)	C25—C26—N2—C27	−16.8 (3)
N1—C11—C16—C15	−178.88 (15)	C32—C27—N2—C26	18.0 (3)
C22—C17—C18—C19	0.6 (2)	C28—C27—N2—C26	−161.96 (15)
C23—C17—C18—C19	−178.91 (14)	O1—C8—O2—C9	−179.46 (16)
C17—C18—C19—C20	−0.5 (3)	C7—C8—O2—C9	−0.95 (18)
C18—C19—C20—C21	0.2 (3)	C10—C9—O2—C8	2.77 (17)
C19—C20—C21—C22	−0.1 (3)	O3—C24—O4—C25	179.48 (17)
C20—C21—C22—C17	0.3 (3)	C23—C24—O4—C25	−2.43 (19)
C18—C17—C22—C21	−0.5 (2)	C26—C25—O4—C24	3.44 (17)

### Hydrogen-bond geometry (Å, °)

**Table:**

Cg2 is the centroid of the C1–C6 ring.

**Table:**

*D*—H···*A*	*D*—H	H···*A*	*D*···*A*	*D*—H···*A*
C16—H16···O1^i^	0.93	2.47	3.305 (2)	149
C31—H31···O4^ii^	0.93	2.58	3.422 (2)	151
C32—H32···O3^ii^	0.93	2.48	3.305 (2)	148
C19—H19···Cg2^iii^	0.93	2.84	3.553 (2)	134

Symmetry codes: (i) *x*, −*y*+1/2, *z*+1/2; (ii) *x*, −*y*+3/2, *z*+1/2; (iii) −*x*, *y*+3/2, −*z*+1/2.
